# Usability testing of the Pathway app: engaging stakeholders to improve access to mental health support for students

**DOI:** 10.3389/fdgth.2025.1633987

**Published:** 2025-11-17

**Authors:** Olugbenga Oti, Sarah Foley, Ian Pitt

**Affiliations:** 1 Insight Centre for Data Analytics, School of Computer Science & IT, University College Cork, Cork, Ireland; 2 School of Applied Psychology, University College Cork, Cork, Ireland

**Keywords:** university students, help-seeking, help-seeking technology, usability, mental health

## Abstract

**Background:**

Mental health difficulties are highly prevalent among university students. Evidence shows that a majority of university students do not seek professional mental health support for their difficulties. Help-seeking technologies provide an opportunity to facilitate students' access to suitable services. This study aims to evaluate the usability of the Pathway prototype, which helps students find support that fits their needs and preferences.

**Methods:**

We conducted three cycles of usability testing with 26 participants comprising students, experts and stakeholders. Students were asked to “think aloud” as they completed tasks using the Pathway prototype. In addition, they were asked to complete the Single Ease Question and rate their experience of using the prototype. Experts and stakeholders were asked to provide feedback following a presentation of the app. The data were analysed using a table of changes approach, thematic analysis and descriptive quantitative analysis.

**Results:**

Following each usability testing cycle, several changes were made to the Pathway prototype. Participants reported that Pathway was a useful app to help students find appropriate support.

**Conclusion:**

In comparing Pathway to current methods of searching for support, students believed that Pathway was quicker, more trustworthy and provided more privacy in searching for support.

## 1 Introduction

Mental health difficulties are prevalent among third-level students [1–5]. According to Lipson et al. [6] and Dooley et al. [7], the rates of depression, anxiety and suicidal ideation among young adults (including college students) have been increasing over the past few years. Research findings indicate that many students are not receiving the appropriate support for their difficulties [2, 8]. Young adults and students are reluctant to engage with mental health support services as a result of barriers such as lack of accessibility (i.e., cost, time and travel) [9–11], lack of awareness of mental health services [9, 10], doubts about treatment effectiveness [9, 10], stigma [9–11], fear of loss of confidentiality [9] and poor mental health literacy (i.e., difficulty recognizing symptoms of mental distress or illness) [9, 11]. Meanwhile, students who have accessed services have experienced long waiting times [12–15], infrequent appointments [12, 13], limited sessions [13], poor referral practices [12, 13], a lack of coordination between services [12], and a frequent change in counsellors/therapists [15].

Help-seeking can be an overwhelming and time-intensive process [16] which is further complicated because students are not always aware of the full range of supports that are available to them [13, 17]. A lack of access to appropriate mental health support could produce negative outcomes, including poor academic performance [18], suicidal thoughts and behaviours [19] and poorer functioning in later stages of adulthood [20].

Help-seeking technologies provide an opportunity to facilitate a student’s access to mental health support services. They facilitate access by signposting users to services, providing mental health education or aiding in symptom recognition [21]. They have been found effective in improving help-seeking attitudes and in increasing help-seeking behaviours and intentions [21]. However, in a recent review of help-seeking technologies, only two out of twenty-one studies mentioned the application of human-centred design, i.e., the inclusion of stakeholders (including end-users) in the development of these interventions [21]. Moreover, only one study, specifically the *Link* program, documented the findings of their user study in the literature [16, 22]. Meanwhile, evidence shows that the inclusion of stakeholders (including end-users) is important for creating technology that reflects stakeholders’ needs and expectations, fits into their everyday lives, is more engaging and more likely to be used [23, 24].

We know of three studies that have documented end-user (students/young adults) participation in the design of help-seeking technologies in the literature. They include the Link program, Thoughtspot and an unnamed web directory [16, 25, 26]. The following text provides a succinct description of these programs.

The Link program aims to facilitate young adults (between 18–25 years) access to mental health support services in Australia. Link users are asked what issues they are facing, how much the issue is affecting them and how they want to access support, e.g., through informational websites, online therapy, face-to-face services, or helplines. Following these questions, users are given a list of national services matching their chosen criteria. They can also view a description of the service, the cost of the service, and what to prepare for when accessing the service. The Link program (now called the ReachOut NextStep tool) has undergone a participatory design process, a feasibility study and a randomised controlled trial [16, 22, 27].

Pretorius et al. [25] designed a website directory for young adults between 18 and 25 years. This directory allows users to view mental health information in various formats, e.g., via podcasts, web pages, training courses, etc. It provides users with information on support services that can be accessed via web chat, helplines, or face-to-face. Finally, it provides users with timely support, e.g., sleep tips or relaxation exercises. This tool is in the prototype stage and has undergone a usability study [25].

ThoughtSpot [28] is a mapping platform that connects young adults (between 16-29 years) with local services in Canada. Students can add “wellness spots” and review existing spots. They can filter services based on hours of availability. In addition, users can view details such as a service description, address and hours of operation. ThoughtSpot has undergone co-design studies, and a randomised controlled trial [26, 28–31].

Participants in the above studies [25, 27, 28] questioned the credibility of the resources/services provided to them. Study participants were mostly female and aged 16–29 years. In addition, in all studies, participants complained that the text was verbose.

These studies provide an opportunity to design a help-seeking tool that prioritises trust and credibility in its resources, caters to a broader range of demographics in terms of age and gender, and is informed by the lived experiences of end-users and the perspectives of different stakeholders.

In addition, while end-user participation has been prioritised in the above studies, the input of other stakeholders is missing. Many eHealth development frameworks advocate for collaboration among various stakeholders (e.g., patients, caregivers, managers, healthcare providers, etc) during the development process of an eHealth program [32]. This is important for identifying stakeholders’ needs and values, and for developing sustainable eHealth technology [32].

Further, only one of the above studies conducted a usability evaluation during the design process [25]. Researchers suggest that usability tests should be comprehensive, including a range of stakeholders and not only end-users [33]. The involvement of multiple stakeholders in a usability test will help gain an understanding of usability issues, generate new requirements for the application, help assess whether the application fits into end-users’ and stakeholders’ way of working and assess the feasibility of the application in solving the intended problems [33].

Usability studies on help-seeking technologies are under-represented in the literature. In addition, we have not found any studies that have documented the involvement of multiple stakeholders in the evaluation of these technologies. The inclusion of diverse stakeholders is important for building trust, commitment and creating technology that is more likely to succeed in the real world [32]. To this end, this paper aims to examine the usability and feasibility of the Pathway app from the perspective of end-users, experts and stakeholders. We present the findings of an iterative usability test conducted with students, experts (psychologists) and stakeholders (e.g., mental health support services). Primarily, we detail the changes made to the Pathway prototype following each cycle of the usability test and provide a thematic analysis of participants’ feedback on various aspects of the prototype. In the following section, we describe the Pathway app.

## 2 Methods

### 2.1 Pathway

**Figure 1:**
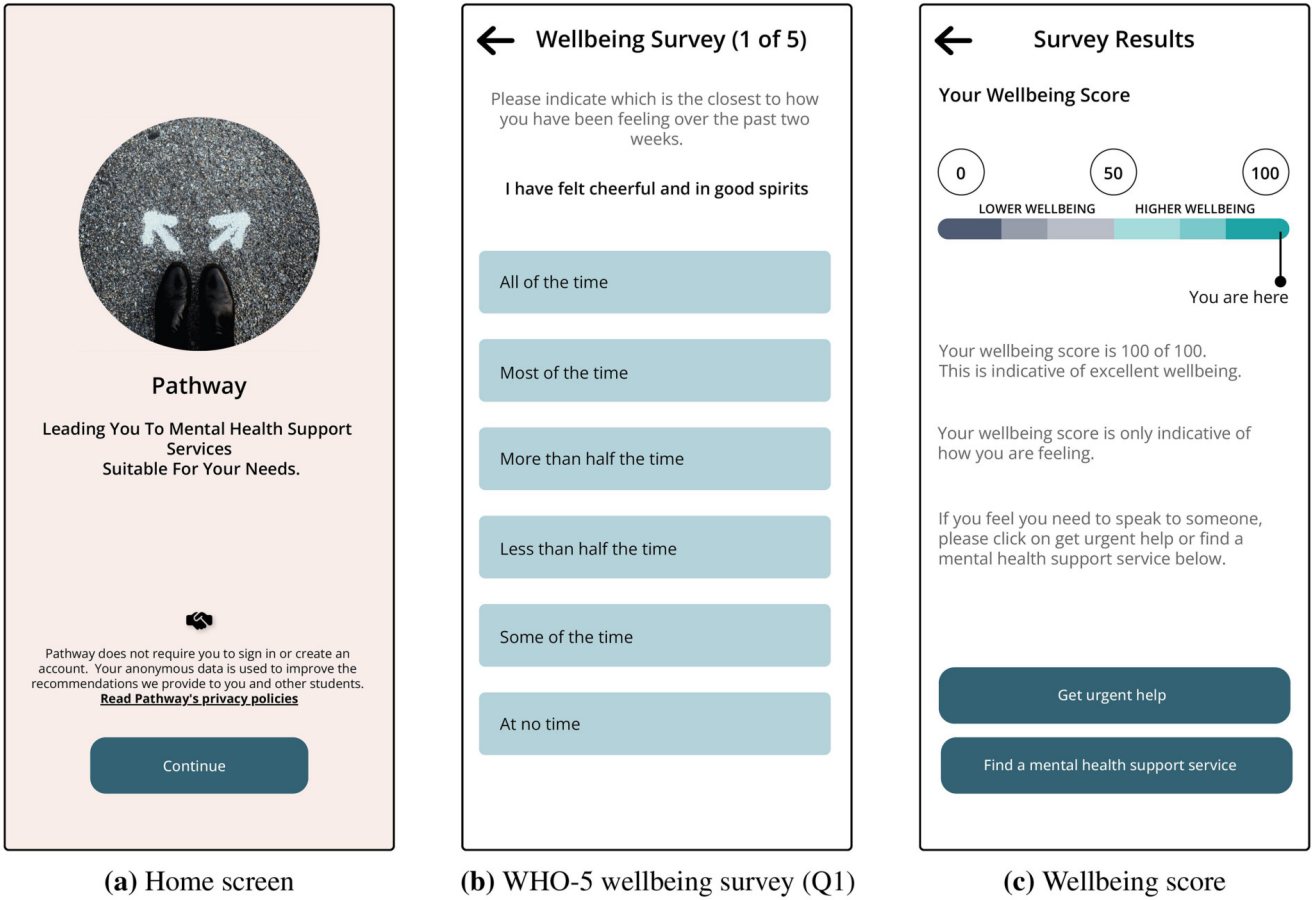
Screens from the pathway prototype. (**a**) Home screen. (**b**) WHO-5 wellbeing survey (Q1). (**c**) Wellbeing score.

**Figure 2:**
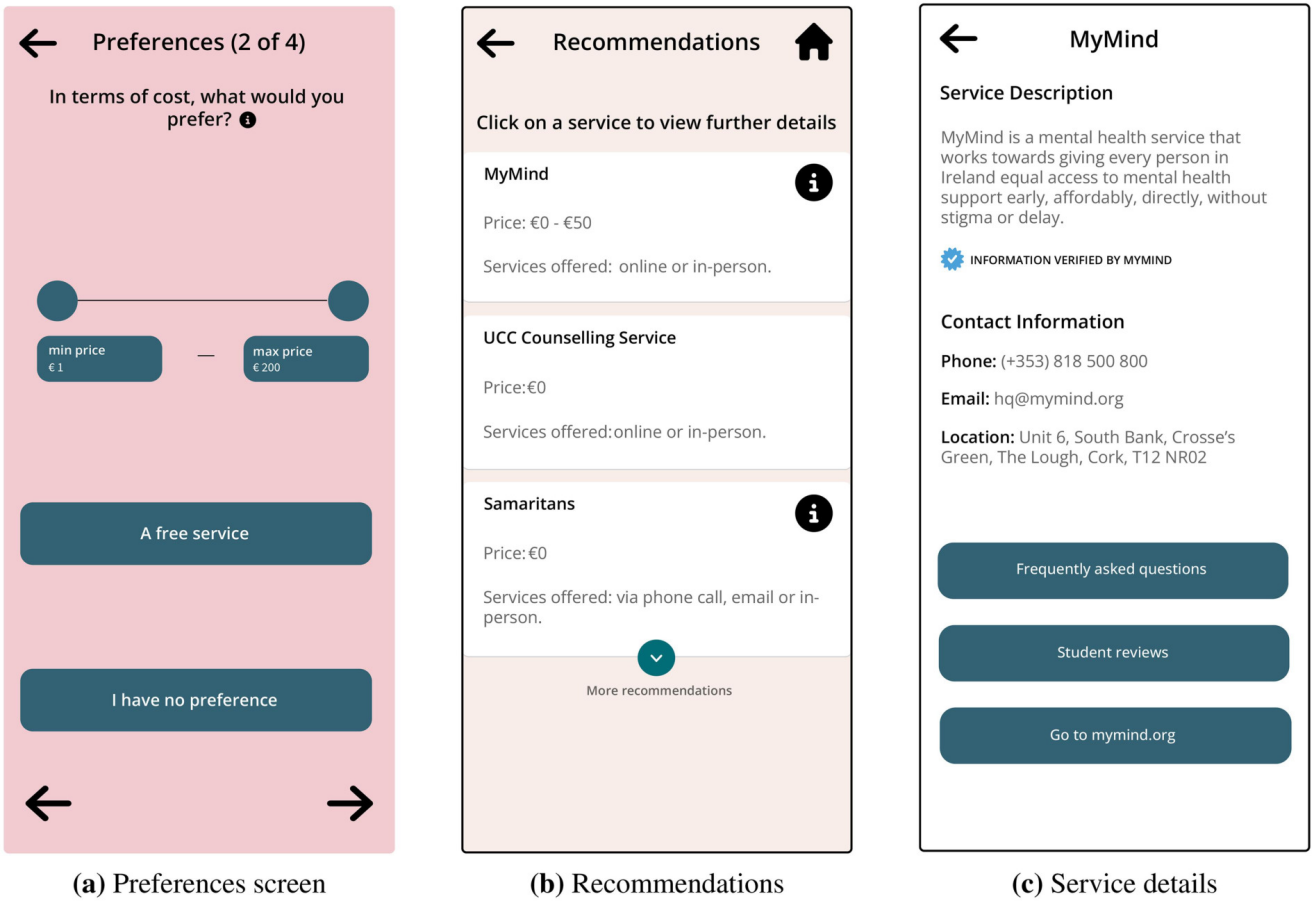
Screens from the pathway prototype (contd.). (**a**) Preferences screen. (**b**) Recommendations. (**c**) Service details.

Pathway, shown in Figures 1, 2, has three main features:

1. It validates students’ need for mental health support through a mental well-being score, which is based on the WHO-5 well-being questionnaire [34].
2. The application allows students to input their preferences (if any) regarding mental health support services. These preferences include a price range, formal (i.e., run by mental health professionals) or informal services (i.e., run by volunteers), counselling/therapy approach (e.g., Cognitive Behavioural Therapy, Person-Centred Therapy, etc.), and format of service (e.g., online or face-to-face services). Following the selection of preferences, it provides recommendations for mental health support services based on their needs and preferences.
3. Finally, upon selecting a service, they will be shown information on that service. This includes a short description of the service, the kind of support they provide (e.g., depression, anxiety, etc), the cost of accessing a service, its opening hours, student reviews, frequently asked questions, a step-by-step guide on how to schedule an appointment with a service and a link to the service website.

An in-depth discussion on the design of Pathway has been reported in Oti et al. [35].

### 2.2 Usability testing

Usability testing is the evaluation of a system by representative users [36]. A usability test aims to ensure that a system is usable (i.e., easy to learn/use) and fits the needs of its target population [36, 37].

Prior to the usability tests, the prototype was tested by two young adults (n=2, between 18 and 30 years old) and an expert in user experience design. Their feedback was used to improve the initial prototype.

An iterative usability test is carried out in cycles, and in between these cycles, the prototype is redesigned based on users’ interaction with the system [38]. The majority of usability problems will be identified by a sample of 8-10 participants per study [36, 39]. In addition, a single test-analysis-redesign can lead to a ten-fold decrease in usability issues [38]. Expert usability tests can reveal issues that remain unobserved in a user-based usability test [37]. In addition, previous research has highlighted the importance of working with stakeholders with expertise in the field of mental health when designing mental health technology [40]. Expert usability tests are usually conducted among those with experience in design or the subject matter the technology is addressing [41]. In this study, we conducted expert usability tests with those with expertise in design and/or mental health provision (henceforth referred to as stakeholders).

The usability objectives for the Pathway program are that (1) it would fit the end-user’s view of seeking mental health support, (2) it would align with stakeholders’ way of working, (3) it would be easy to learn, use and navigate, (4) it would be perceived as simple and visually appealing, and (5) the language would be clear.

### 2.3 Ethics

We received ethical approval from the Research Ethics Committee of the relevant universities under log numbers UCC-2023-048A4 and MTU-HREC-MR-23-043A.

### 2.4 Inclusion and exclusion criteria

#### 2.4.1 Students

The inclusion criteria for students were (1) aged 18 years or older and (2) currently a student at University College Cork (UCC), Ireland or Munster Technological University (MTU), Ireland. Students who did not fit the above criteria were ineligible to participate in the study.

#### 2.4.2 Experts

The inclusion criteria for psychology experts were (1) experience in the design of mental health technology and (2) employed at the Department of Psychology at University College Cork, Ireland or Munster Technological University, Ireland.

#### 2.4.3 Stakeholders

The inclusion criteria for mental health stakeholders were individuals or organisations that were involved in signposting students to mental health support or providing students with mental health support. This included those in managerial positions who aligned with the same goals. These stakeholders include representatives of the health service executive in Ireland, the student union at Irish Universities, psychologists and psychotherapists, youth mental health support services, the Higher Education Authority, and GPs and Counsellors at Irish Universities.

### 2.5 Study advertisement

#### 2.5.1 Students

Students could sign up for the study through a landing page where they could submit their email addresses to signify their interest in participating in the study. The study was advertised to students at UCC and MTU via the X platform, and students could access the landing page through the post. In addition, posters leading students to the landing page were placed around the UCC campus. Students in UCC were also recruited through university-wide mailing lists. Further, undergraduate/postgraduate students in the department of Computer Science and undergraduate students in the department of Psychology and Computing were contacted via their departmental mailing lists. The authors of this paper have a connection to at least one of the listed departments. Finally, the student affairs department and the student union were engaged to recruit students from MTU.

#### 2.5.2 Experts (psychologists)

Staff who have experience in the design of mental health technology and were employed at the Departments of Psychology at MTU/UCC were contacted directly via email. In addition, experts who had participated in the study were asked for recommendations of people with similar expertise, meeting the same criteria.

#### 2.5.3 Stakeholders

Stakeholders were contacted via email and through mailing lists of relevant professional bodies, for instance, the Psychological Society of Ireland and the Irish Student Health Association. In addition, posters were placed on the X platform. Further, a member of the research team is employed by the National Suicide Research Foundation in Ireland and therefore has a working history with these stakeholder groups. Therefore, they were able to introduce several groups to the research project via email.

### 2.6 Informed consent

All participants provided informed consent through consent forms, and they gave consent for the information provided to be used in research publications. The consent was provided by signing a consent form.

### 2.7 Design and procedure

The following subsections present the design and procedure for the usability studies involving students, experts and stakeholders.

#### 2.7.1 Students

We conducted a remote moderated usability test using Microsoft Teams. The usability tests were video-recorded and lasted a maximum of an hour. Students were provided with scenarios focused around Pathway’s three main features and were asked to “think-aloud” (i.e., verbalise their thoughts ) as they completed the tasks. These tasks are included in the appendix.

Students were asked semi-structured interview questions after completing each task and at the end of the usability test. Semi-structured interviews are a useful way of getting subjective feedback from participants on their experience with the prototype [37]. The questions (included in the appendix) centred around the completion of the task using Pathway, the privacy of data within Pathway, current methods of searching for support, the perceived usefulness of Pathway, and features that might encourage students to use the application.

Further, students in the third cycle of usability tests were asked to complete the Single Ease Question after completing each task. The Single Ease Question asks the participant to rate the difficulty level of the task they just completed from Very Difficult to Very Easy on a 7-point scale. The Single Ease Question points out usability issues in different parts of an application [42]. At the end of all tasks, in both cycles, students were asked to rate their experience using the Pathway application on a 5-point scale, where 1 is “very dissatisfied” and 5 is “very satisfied”.

#### 2.7.2 Experts (psychologists)

Experts who agreed to participate in the study were sent a four-page PowerPoint presentation before the study; this helped them become further acquainted with the study. This presentation included a problem statement, the features of Pathway and some concluding remarks. We conducted a remote expert usability test using Microsoft Teams. The usability tests were video-recorded and lasted a maximum of an hour. The usability test began with a brief background of the studies that led to the design of Pathway, followed by a presentation of the Pathway prototype. The presentation lasted about 10 min. Afterwards, experts were asked semi-structured interview questions. The questions (included in the appendix) encompassed general opinions on Pathway, its feasibility in supporting students seeking support, features of Pathway and the language used in Pathway. At the end of the study, experts were also allowed to provide further comments related to the Pathway app or help-seeking among students.

#### 2.7.3 Stakeholders

Stakeholders participated in the study via focus groups or interviews on Microsoft Teams. Focus groups and interviews were video-recorded and lasted approximately 90 min and 60 min, respectively. The expert usability test began with a brief background of the studies that led to the design of Pathway, followed by a presentation of the Pathway prototype. The presentation lasted about 10 min. After the presentation, stakeholders were asked to provide feedback on the Pathway prototype. The prototype remained on screen while stakeholders provided their feedback, and they could ask to see different aspects of the prototype. This expert usability test was conducted as part of a larger study in which stakeholders discussed student mental health support needs, barriers students face when seeking mental health support, barriers stakeholders face in addressing student mental health needs, challenges of integrating the Pathway application and opportunities for collaboration. Findings related to challenges/recommendations for the integration of Pathway are reported in a separate paper [43].

### 2.8 Pretesting

Before the usability tests, a dry run of the study was conducted with two young adults and an interaction design expert. The testing aimed to prepare for the usability studies and improve the prototype. Both pretest sessions began with a brief presentation of the studies that led to the design of Pathway. The two young adults were engaged in a think-aloud session as they completed the same tasks as students in the first cycle of usability tests. They were also asked to answer the same semi-structured interview questions. The session was conducted on Google Meet, lasted approximately one hour, and was not recorded. A brief presentation of the Pathway prototype was provided to the interaction design expert. This was followed by the opportunity to provide free-form feedback on the prototype and the language used within the prototype. The session was conducted on Google Meet, lasted approximately one hour and was not recorded.

As shown in Table 1, participants’ comments encompass interface design, prototype functionality and accessibility. They suggested adding a link to more information on the WHO-5 wellbeing survey to give users more details and context. They suggested changing the button text for the wellbeing survey from “Take the mental health survey” to “Get my mental wellbeing score”. They noted that this text change would make the survey more engaging to users. Participants suggested replacing the delete buttons for recommended services with thumbs up/down buttons to prevent users from accidentally removing recommendations. In addition, they suggested providing a brief explanation of each therapy approach listed, as this would support the user’s decision-making. Finally, participants suggested including information on emergency hotlines on the home page and increasing the hierarchy of the urgent help button. These changes aligned with the usability requirements (i.e., the application should be useful in urgent situations) for the prototype [35]. All changes are listed in Table 1.

**Table 1:** Changes following pretest sessions.

Interface design	Functionality	Accessibility
Added link to information on WHO-5 wellbeing questionnaire		
Added a pop-up for counselling approaches		
Replaced delete icons with thumbs up/down icons on recommendation screen	Made buttons in preferences section clickable	
Changed position of urgent help to the top of the button list	Added information on Samaritans	Changed button outline to grey to improve colour contrast
Changed button text for survey to “get my wellbeing score”	Added information on emergency hotlines	
Used pictionary style design for therapy approaches	Added brief description of each therapy approach	
Added vertical margin to buttons		

### 2.9 Analysis

The video recordings from the usability tests were transcribed. These transcripts were analysed using an adapted version of the Table of Changes method in the person-centred approach [44]. We compiled negative, positive and neutral comments about the Pathway prototype on a Miro board. We also noted the changes made to the prototype after each cycle of usability testing. The Table of Changes method [44] was used after each cycle as a quick way to organise participants’ feedback and identify potential changes.

In addition, we conducted a thematic analysis of participants’ feedback on various aspects of the application. The analyses were conducted in NVivo version 1.5.1. The thematic analysis provided a more in-depth evaluation of participants’ feedback on the Pathway app. We conducted a descriptive statistical analysis of participants’ ratings of their experience in using the Pathway prototype and their responses to the Single Ease Question. Using multiple methods to evaluate the system can help gain a more in-depth understanding of how users perceive the system [45].

#### 2.9.1 Reflexivity-thematic analysis

The six phases of thematic analysis by Braun and Clarke were followed, i.e., familiarisation with the data, coding the data, generating initial themes, developing and reviewing themes, refining themes, and writing up the report [46]. After the study’s completion, the first author transcribed the data, which enabled familiarisation with the data. In addition, initial analysis to consider potential changes to the prototype also built familiarisation and supported immersion in the data. The first author conducted an inductive coding process, developing codes resembling the content of the data. The codes were then organised into these themes. These themes were semantic, bearing close similarities to the interview questions and features of the application. These themes were important areas for consideration in the design and development of the Pathway app. Following the refinement of the theme names and content, the first author wrote the report. This study aimed to improve the prototype design of the application, Pathway. Therefore, our focus in the thematic analysis was to identify areas for improvement and summarise participants’ feedback on issues surrounding the development of the app, for instance, anonymity and privacy. Therefore, the themes developed here are topic summaries of these identified areas as opposed to a shared meaning around a central concept [47]. This makes the thematic analysis more akin to codebook approaches. A similar conceptualisation of themes can be found in Clarke et al. [48]. Finally, we note that this is an inductive thematic analysis with a realist approach, meaning that we have taken participants’ feedback and opinions as objective truths.

## 3 Results

### 3.1 Participant characteristics

A total of 26 participants took part across three usability testing cycles:

1. The first cycle was conducted with five students (2 females, 3 males, mean age = 25.2 years) and two psychologists at the university.
2. The second cycle was conducted with 14 stakeholders within a series of focus groups and interviews
3. The third cycle was conducted with five students (2 males, 3 females, mean age = 29 years).

Full demographic information was collected from students to show the diversity of participants. However, for stakeholders and experts, their professional expertise was deemed most important. Table 2 shows the demographic information of all participants.

**Table 2:** Demographic characteristics of participants.

Cycle	ID	Participant type	Course of study/Job title	In-text^a^^,^^b^ Identifier	Gender^b^	Age^b^	Year^b^ of Study
**1**	1	Student	BA Sociology and Economics	C1, P1	Male	20	2
	2	Student	BSc Accounting	C1, P2	Female	22	4
	3	Student	PhD Computer Science	C1, P3	Male	25	1
	4	Student	PhD Computer Science	C1, P4	Male	40	4
	5	Student	BA Psychology and Computing	C1, P5	Female	19	2
	1	Expert	Psychologist with experience in the design of mental health technology	C1, E1			
	2	Expert	Psychologist with experience in the design of mental health technology	C1, E2			
**2**	1	Stakeholder	Student union officer at Irish University				
	2	Stakeholder	Employee of the Health Service Executive				
	3	Stakeholder	GP at Irish University				
	4	Stakeholder	Student counsellor at Irish University				
	5	Stakeholder	Employee of Higher Education Authority in Ireland				
	6	Stakeholder	Student union officer at Irish University				
	7	Stakeholder	Clinical manager at mental health support service				
	8	Stakeholder	Psychologist in private practice				
	9	Stakeholder	Student union officer at Irish University				
	10	Stakeholder	Psychotherapist in private practice				
	11	Stakeholder	Director of youth mental health support service				
	12	Stakeholder	Representative of youth mental health support service				
	13	Stakeholder	Psychologist in private practice				
	14	Stakeholder	Employee of the Health Service Executive				
**3**	1	Student	PhD Computer Science	C3, P1	Male	40	4
	2	Student	PhD Computer Science	C3, P2	Female	32	3
	3	Student	PhD Applied Psychology	C3, P3	Female	25	3
	4	Student	BSc Computer Science	C3, P4	Male	23	4
	5	Student	BA Psychology and Computing	C3, P5	Female	25	1

a Note: For stakeholders, job title is the same as the in-text identifier.

b If applicable.

### 3.2 Table of changes

As mentioned earlier, following each cycle of usability testing, participants’ feedback was organised into positive, negative and neutral comments. Based on these comments, changes were made to the prototype following each cycle of usability testing. Table 3 shows the changes to various aspects of the prototype across the three cycles of usability testing.

**Table 3:** Changes following each cycle of usability tests.

Cycle	Navigation	Interface design	Functionality
1		Increased font size	Added more information on WHO-5 wellbeing survey
		Centered text	Amended the description of the Pathway app
		Increased spacing on pages	Added more information on the 50,808 service
		Amended link for WHO-5 wellbeing survey	Added intro. page for WHO-5 survey
		Changed colour scheme for each section of Pathway	Added urgent help button on survey results page
	Added headings to pages	Added visual interpretation of survey results	Added information on typical cost of therapy, average length of therapy, and average duration of each session
	Changed close buttons to back buttons	Improved visibility of link to further information on services	Clarified that Pathway only provides a recommendation service
	Added home icon to pages	Changed design of service details and FAQs	Removed thumbs up/down on recommendation screen
	Changed button colour to maintain consistency	Moved link to service website for increased visibility	Added addresses, phone number and email of services
	Removed forward button to reduce number of clicks	Harmonised font and font sizes to maintain visual hierarchy	Clarified sources of information for service details and reviews
		Removed quote from student union on survey results page	Added functionality for users to submit reviews
2			Removed preference for counselling approaches
			Added preference for location
		Made wellbeing score bold for emphasis	
3	Removed broken link on splash screen	Added information icon to improve visibility of links on recommendation screen	
	Fixed incorrect navigation on urgent help page	Fixed FAQ drop-down	

### 3.3 Thematic analysis

Following our study of the data, we outlined eight themes which detail participants’ feedback on various sections of the application and their perspectives on the issues surrounding the development of the app. These themes are: (1) Pathway’s description, (2) anonymity and privacy, (3) WHO-5 well-being survey, (4) interface design, (5) user preferences, (6) recommendations page, (7) service details, and (8) students’ choice of services. The themes are described in the following subsections. Participants are denoted according to the cycle and participant number. Experts are denoted as E. Stakeholders are denoted by their ID and job title. Table 2 shows information on all participants.

#### 3.3.1 Pathway’s description

Due to the proliferation of mental health apps, we considered it important for users to easily understand the purpose of the Pathway app. We posited that this would help them decide if the app suited their needs. This theme summarises participants’ understanding and misunderstandings about the purpose of the Pathway app. Through the description of Pathway provided, participants understood that it was not suitable for emergencies:

I think this is a really important kind of initial message. So you know, if it is a very urgent emergency that they have. A sense that maybe the app is not the space for them, but actually they need to call the emergency helpline. [C1, E1]

In the above quote, the expert noted that having this message “If you feel you are a danger to yourself or others, please call 112 or 999” on the home screen was important for directing those experiencing emergencies to the right service.

Participants thought that they needed to complete the WHO-5 wellbeing survey before being directed to mental health support services:

…for me, it looks like in either of the ways I need to first score (Get my mental well-being score) and then like to get some support [C1, P3]

The description of the features of Pathway on the home screen was unclear to participants. They believed that they needed to get their mental well-being score before finding a mental health support service. These comments led to an improvement of the prototype by describing each feature of Pathway on a separate screen using a card-style design. Following these improvements, the description of Pathway became clearer to participants. In the next theme, we summarise participants’ feedback on anonymity and privacy in mental health apps (including Pathway).

#### 3.3.2 Anonymity and privacy

This theme summarises participants’ perceptions of privacy and anonymity within Pathway and other mental health apps. Participants indicated that they appreciated not needing to sign in to the Pathway app. They noted that this was especially important in a mental health related app.

I like that it’s anonymous and anonymous data and you don’t need to sign in. [C1, P1]

That’s perfect for stuff like this, especially when it comes to more touchy subjects. [C3, P4]

In the above quotes, participants discuss their appreciation of the privacy policies within Pathway, specifically, “Pathway does not require you to sign in or to create an account. The anonymous data you provide is used to improve the recommendations we provide to you and other students.” They felt it was important for a mental health related app to offer this level of privacy to its users.

Although participants appreciated being able to use the app anonymously, they considered the implications of not creating an account. They made comments like “if I wanted to use Pathway again, would I have to start again?”, “I’d kind of like to be able to kind of go through like my past experiences on the app”, “with that the well-being one as well. I think it would be good to have a track record”. Others mentioned that they did not mind repeating certain tasks to maintain their privacy:

So you do have to trade off sometimes with them—that ease of access vs. privacy. So sometimes you can’t do too much about it.. [C1, P4]

This implies that while many users may appreciate using Pathway anonymously, others might desire features that are available in a non-anonymous setting.

When asked what they would expect from the privacy policy for Pathway, participants mentioned that they would expect the privacy policy to be “straightforward” and that their data would not be “used for any type of marketing”. The following comment exemplifies this case:

If I input anything, which of them they’re keeping and what they’re using it for …I’d like to see something like what you input for this is only used for this, that like it’s not taking any other information like location and stuff like that. I always appreciate [it if] it’s laid out very clearly. If it’s like understandable [in] layman’s terms, and because very often they’re kind of, they put fancy words in it to make it less understandable…So I think if I read the privacy policy and it was very clear to me what was happening, I would immediately trust the app more. [C1, P5]

The above quote implies significant experience with privacy policies and clear expectations for privacy policies in a trustworthy app. This is not unsurprising for a digital native with experience in mobile apps and other technologies.

This theme suggests that participants appreciated being able to use the Pathway app anonymously. However, they also understood the implications of maintaining their anonymity, for example, the need to repeat certain tasks. This was described as a tradeoff. Finally, participants displayed a good command of their privacy needs, for example, that a privacy policy should be straightforward and include details on how their data is used. In the next theme, we summarise participants’ feedback on the wellbeing survey included in the Pathway app.

#### 3.3.3 WHO-5 wellbeing survey

This theme depicts participants’ experiences of completing the WHO-5 well-being survey. They described their perceptions of the survey and made suggestions on how to improve the presentation of the results.

Participants were asked to fill out the WHO-5 well-being survey using the Pathway prototype, and they were instructed to answer every question with the option, “All of the time”. The survey was described as “simple”, “easy to read”, “short” and “positively-worded”. Participants in the first cycle of usability tests highlighted that a “visual indication” of the survey results would be useful. A graph depicting the score was added to the prototype.

Participants indicated that they liked the graphic [in Figure 1(c)], especially that the colour for lower wellbeing was not red. They also appreciated that the interpretation of the score did not provide a diagnosis. The following comments exemplify this case:

[The graphic is] a nice way of representing it [referring to the wellbeing score], definitely, and I guess actually I kind of like that there’s no red for the lower well-being in that you know it’s not like danger kind of indicating, I think yeah, it’s displayed nicely right now. [C3, P3]

And I understood like it’s just scoring how you’re feeling.. it’s not saying you have depression or anything like that, it’s just scoring how you’re feeling. [C3, P5]

In addition, participants felt that the interpretation of the score was supportive because it led them to mental health support services even when they had a good wellbeing score:

You know, it’s [not] kind of like…good, you’re great, you can go, Goodbye. It’s saying, you know, it might be a good score, but if you feel bad. Yeah, it’s been kind of fairly cautious and careful in making sure that you know, they know the options and if they do feel bad, even still that they should talk to somebody. [C1, P4]

Stakeholders expressed concern about the presentation of low wellbeing scores to users. Therefore, participants in the final cycle were asked how they would like a low wellbeing score to be presented to them. Participants suggested either an objective approach or a suggested one. These comments exemplify these cases:

I think it could be like a suggestion. Like maybe it seems like you’re having a difficult time [C3, P2]

I think that giving the data in the most like hands-off way so like…Yeah, like [you have ] low well-being. I think that’s the best way to do it. [C3, P4]

In the above quotes, participants suggest two distinct approaches to presenting a low wellbeing score. This is an area for further research into what approach increases the likelihood of further help-seeking.

In this theme, participants indicated that they appreciated the graphical representation of the survey results, that they were not provided with a diagnosis and that they were directed to mental health support services. Finally, participants indicated that they would want the tone of the results to be either objective or suggestive. The next theme explores participants’ perceptions of the user interface and design of the Pathway app.

#### 3.3.4 Interface design

In this theme, participants discuss the usefulness of the Pathway app, its user interface and the language used. They also note areas in need of improvement.

Participants’ impression of the Pathway app was that it is “friendly”, “nicely laid out”, “simple to use”, and is “clearly aimed at students”. In addition, they noted that the “presentation of the language [was] really clear” and it was “not overwhelming” to the user.

In certain parts of the application, participants indicated that the text was “too small”. However, following improvements to the prototype, participants in the final cycle noted that the text was “a good size”.

Participants mentioned that they liked the colours in Pathway. They described them as “calm” and “approachable”. Nonetheless, they felt that the colour variations were “confusing”. They suggested using colour variations to “highlight the different services” within Pathway. This change was enacted in the final cycle.

While some participants appreciated having two clicks before moving to the next question, others found it tedious. The following comments exemplify these issues:

I like [doing] the clicking and then clicking on because it does allow you to kind of think about what, like if I was putting thought into this, I’d like to see what I chose and then move on and the ability to go back is also quite comforting, especially because it’s more of an important one [referring to the wellbeing survey]. [C1, P5]

Yeah, for me it’s irritating. Click twice for the same thing. I rather just if I make my selection, you move to the next option. [C3, P2]

The above quotes represent another distinction in participants’ experiences of certain design decisions. While some appreciated being able to think through their responses (in the wellbeing survey) before deciding to move to the next question, others felt that it was unnecessary to have two clicks. This is an area for further exploration to see which option produces a higher likelihood of encouraging progress in help-seeking. Finally, participants found the consistency in design patterns within the application to be helpful:

I think that consistency really helps to kind of remember how to do things and to redo things, maybe that you’ve already done [C3, P3]

During the usability tests, participants were asked to repeat the task of finding a mental health support service. The purpose of repeating the task was to observe their ability to navigate and remember how to complete the task. The above quote indicates that consistency within the Pathway prototype made it easy to complete this task a second time.

This theme implies that participants appreciated the design decisions made in the Pathway app, for instance, the layout, language, colours and consistency in the design patterns. The navigation pattern, specifically the number of clicks in the well-being survey, is an area for further research. The next theme explores participants’ perceptions of the preferences section of the Pathway app.

#### 3.3.5 User preferences

As mentioned earlier, Pathway allows its users to select their preferences regarding mental health support services. These include cost, formal/informal services, counselling/therapy approach and format of service (e.g., online or face-to-face services). This theme presents participants’ feedback on the preferences survey, i.e., what they appreciated and areas for improvement. Participants appreciated that the questionnaire was “only four questions” and they noted that the questions were useful to those seeking support:

I thought they were clear, and I understood why they were being asked, which I think is very important for any kind of questionnaire. And yeah, I do think that they would represent what people want. I do think it’s important to get people thinking about what they actually want because that might not have been something someone even considered. [C1, P5]

The above quote indicates that the questions asked align with students’ needs regarding mental health support. The participant also believed that it would help students consider what their preferences are regarding mental health support.

Participants highlighted that Pathway did not provide adequate information on the price range for therapy, and they noted that this preference was not suited to potential first-time users of therapy:

I do not know, like [at] this stage, first I do not know is it price for one session? Is it [the] price for solving my needs? Second, if it’s [the] price for one session, before [the] actual support, how do we know how long the full course will be? How many sessions? So it’s hard to like calculate [the] budget in advance here. [C1, P3]

Information on the typical cost of therapy in Ireland, the average length of therapy, and the average duration of a session were added to the prototype. In the final cycle of usability testing, a participant stated:

…I like the extra information that you get here (Information button on cost), and like I know they average around 80e, but it’s nice to be told again. [C3, P5]

Experts expressed doubts about students’ familiarity with the different types of therapy approaches and their ability to “know what therapy suits them”. Some students thought it was beneficial to have this information included in Pathway, while others felt it could be overwhelming:

Like the first time I ever looked at all the different types of therapy you could possibly go through, I was like just overwhelmed so…And like it was, it was actually a little bit of a turn-off for me because I was about like 19 and I was going for it myself, like I wasn’t consulting my parents, any other family members, any friends? I was just going it alone. So it was like really like Googling a lot and trying to figure out what everything meant. [C1, P2]

The above quote gives a window into young adults’ experiences of seeking help for the first time. The participant indicates that they were very young and looking for support on their own. They were also overwhelmed by the amount of information they received when conducting a web search. This quote further emphasises the need for a simplified entrance to therapy through an app like Pathway. It is also important that such a tool caters well to first-time users of therapy without overwhelming them.

Stakeholders had differing views on the inclusion of the therapy approaches, and some agreed that it would be overwhelming for students. Others highlighted that it was important to teach students about therapy approaches, for instance, “what’s the best therapy for X”, expected “frequency of appointments”, and the “commitment involved in terms of time and home practice tasks”. The preference for therapy approaches was removed from the prototype in the final cycle of testing. However, a future version of Pathway will include an education feature which provides information on therapy and therapy approaches.

Participants and stakeholders suggested adding a preference for location “because some places might be easier to get to than others”. This is especially important in the Irish context as the public transportation is often unreliable [49]. This preference was added to the prototype.

Participants noted that it was beneficial to have various ways of communicating with a support service:

Yeah, this is pretty useful as well, I think. Just I know from hard times, you know, you might not have your phone topped up or something, so having the e-mail option and then as well vice versa, like we [may not have] Wi-Fi, so might have to use the calls or messages. [C1, P1]

Finally, participants wanted Pathway to include a preference for group therapy:

I’ve actually been kind of looking around for support groups for like different types of things. But I’ve never really found them. I think I would like to see that here as well. [C1, P2]

This feature will be added to a future version of Pathway.

The preferences survey aims to narrow participants’ choices of services. Participants appreciated the usefulness of the survey for those seeking support. However, they suggested including more information on therapy and therapy approaches. In addition, they suggested including preferences for location and group therapy. In the next theme, we explore participants’ perceptions of the recommendations page.

#### 3.3.6 Recommendations page

On the recommendations page, participants were presented with three mental health support services along with a drop-down through which they could view more services. There was also a thumbs-up and thumbs-down beside each service to indicate if they liked/disliked the service. In this theme, participants provide suggestions for improving the features and the user interface of the recommendations page.

Participants found it difficult to interpret the meaning of the thumbs-up/down feature. They expected that it would be visible after they had used the service:

I would probably expect to find these after I found this [referring to the recommendations page] or after I’ve used the service. So if you click into the Samaritans and then you engage with them and then you kind of after you finished on this app or whatever, then you would expect maybe did the service work or not or something like that you know? [C1, P4]

The purpose of the thumbs-up/down feature was to help users indicate services that they liked/disliked based on past usage and prior to their use of Pathway. The thumbs-up/down feature was removed from the prototype because it was confusing to users. Meanwhile, users can submit reviews of services they have attended through Pathway.

Participants did not perceive the clickability of the service element [see Figure 2(b)], as the following comment illustrates:

I’m not sure if there is, but if there’s like a link to more, oh there is! I was just about to ask you if there was like a link to more information about the services…But yeah, even just like a small icon like a little ’I’ next to it, I think could maybe be really, really useful just to signpost that. [C3, P3]

An information icon was added to the prototype, thereby enhancing the element’s clickability.

Finally, participants were interested in information on the waiting times for the services:

Another thing could be availability umm cause if I see one and that has a waiting list of two years [I’m] probably not gonna go for that one [C1, P5]

Information on waiting lists will be added in a future version of Pathway.

After a service is selected on the recommendations page, a user is presented with detailed information on that service. In the next theme, we present participants’ feedback on that section of the Pathway app.

#### 3.3.7 Service details

This theme summarises participants’ feedback on the information Pathway provides about a service, specifically, the description of the service, contact information, FAQs, and student reviews.

Participants appreciated that the information within Pathway was verified and that the sources of information were clearly stated:

Verified by [the] service, I would like that. Umm, it makes me feel better about it. [C1, P5]

And I think the idea that you’re giving sources for your information is really nice and really important for students to see where the information is coming from. [C1, E1]

Students felt more confident in the information because it was verified by the service. In addition, students and experts appreciated knowing the sources of the information presented within Pathway. Experts felt it was especially important in the age of fake information.

Participants noted that the frequently asked questions “[answered] all of the questions [they could] imagine”. In addition, it helped distinguish between the support services, for instance, one participant stated “I think the questions, the FAQs, they’re really good and just show the difference, what the difference is between the [services], Samaritans and MyMind.” Students and stakeholders found the question “what are my next steps” especially useful, for instance, a clinical manager at a mental health support service stated:

I think what is very good is that it reduces that uncertainty of what is going to happen, like, yes, there are at service, but how does the service work and what do I have to expect from booking an appointment, this is really, really good. I think that it would be very helpful and reduce that barrier of actually engaging with the service. [7, Clinical manager at a mental health support service]

This implies that even when students are aware of a service, not knowing what to expect from a service or how to book an appointment can be a barrier to accessing that service. By providing students with this information, Pathway addresses that barrier to help-seeking.

Students were generally positive about having access to reviews and the ability to submit reviews. However, stakeholders and experts expressed concern about skewed reviews and the effects of negative reviews on students, counsellors and therapists. For instance, a GP at an Irish University stated:

…if you have feedback that comes from a survey of all people who are engaging with the service, I think that I’m a bit happier with that. I’m very wary about isolated reviews because I know that sometimes those can be skewed if you go online and look at reviews, people who are very happy with something, they’re not as motivated to go online and type a review as someone who is dissatisfied…I’m just worried that someone who could be a good counsellor or a good doctor, that they just, you know, there was just something in that one-on-one personal interaction that then gets painted as that’s not a good service. [3, GP at Irish University]

In the above quote, the stakeholder felt that reviews are more likely to be balanced if they are collected from a cohort of service users rather than submitted by individual users. The lack of consensus between stakeholders and students on reviews, represents an area for further research.

Finally, participants expressed a desire to have concrete information on how privacy is maintained within a service:

So I definitely wanna find out more about the like, how they keep the conversations private? ’cause I know they have like a lock-box or so, somewhere they store the files away. So I wonder, like for them, what is their kind of protocol for that as well? [C1, P2]

This information was not readily available on the service website that this participant was browsing through. There is a need for services to be transparent about how privacy is maintained within a service. In a future version of Pathway, support services will be asked to provide this information to users.

The comprehensive information Pathway provided helped support participants’ choice of support services. In addition, the verification of information and citing of sources supported credibility and trust. Participants implied that in doing so, Pathway set itself apart from other tools they had used. In the next theme, participants distinguish their experience of using Pathway from other tools, such as a web search.

#### 3.3.8 Students’ choice of services

Across the feedback, participants compare their experiences of using Pathway to their current methods of searching for support. Students were asked how they would search for mental health support in the absence of Pathway. They indicated that they would use “Google”, or ask their “friends” about suitable services. They noted that Pathway was quicker, more personal, and more trustworthy than using a web search. They believed it would be a “lot more helpful in getting” them to their “end goal of getting help.” In addition, participants mentioned that their use of Pathway had taught them about the different types of mental health support services and that through the usability study, they had become aware of services that were previously unknown to them. The following quotes exemplify these cases:

I think Pathway is easier because it will give me a list of so many options. And also the way it is formulated, it’s in a way that I can know, OK, actually there are formal services, there are informal services, somehow it just also give[s] me some awareness. There [are] some paid ones, [and] there are free versions. It helps you to give you a starting point, a good starting point. [C3, P2]

Like, because again, I’ve never heard of MyMind or Samaritans knowing that they are a service now is going to be beneficial to me or anyone who I interact with. I’ll just tell them about it. So yeah, I found it good [C3, P5]

The above quotes indicate that Pathway provides a solid foundation to those seeking mental health support. In addition, it implies that Pathway’s usefulness extends beyond the user to the user’s social network. Further, participants described how Pathway could support their decision on appropriate services. For instance, they noted that they would choose formal or informal services depending on their WHO-5 well-being score:

…if I got a low score on the feelings, I’d probably go formal service. Well, maybe if I got an average or high score, but I still feel like I need help, I’ll go informal. [C3, P5]

Finally, participants explained how different information within Pathway would guide their choice of services. In the following quote, a participant describes how price per session, reviews, opening hours and frequently asked questions would inform their choice of services:

For myself, and while I’d look at pay immediately with this one, I like the idea of a sliding pay scale, and because it also makes me like the service more and that they have things like that and…the opening hours I would use as well because I’d have to go around college and. I would look through all the reviews and make sure no one’s saying anything that sounds awful and I would look at the frequently asked questions. There’s enough information that I feel like I’m getting fully informed. [C1, P5]

In describing their experiences using Pathway, participants noted a clear advantage to using this tool over their current methods of searching for support. They noted that Pathway was quicker, more personal and trustworthy. They mentioned that Pathway had taught them about services, and types of services, and could support their choice of appropriate services. For example, in terms of price per session or wellbeing score.

### 3.4 Quantitative findings

In the first and third cycles of the usability tests, students were asked to rate their experience of using the Pathway prototype on a scale of 1 to 5, where 1 is very dissatisfied and 5 is very satisfied. Five participants in the first cycle rated their experience, with an average score of 3.8. Following improvements to the prototype, participants in the final cycle rated their experience as 4.3(n=4). P1 was excluded from the statistical analysis because they encountered system-specific challenges with the prototype that could not be replicated and were not experienced by any other participants.

In the third cycle of the usability tests, students were asked to complete a single ease question following their completion of each task. Table 4 shows the average scores of the single ease question (on a 7-point scale) provided by participants in the third cycle of the usability test. The task “Find a mental health support service using Pathway” was the most difficult to complete. This was because participants could not perceive that the services were clickable. This was rectified during the final cycle using an information icon.

**Table 4:** Responses to single ease question (Cycle 3).

No	Tasks	Overall this task was? (n=4)
1	Completing the mental health survey using Pathway	7
2	Finding a mental health support service using Pathway	6.25
3	Using Pathway to select preferred service	6.5
4	Using Pathway to find a new service	6.5

## 4 Discussion

### 4.1 Summary of findings

In this study, we explored the usability of the Pathway prototype with university students, psychologists and stakeholders. We conducted iterative usability testing with 26 participants across three cycles. Based on participant feedback, we made changes to the prototype following each cycle of usability testing. These changes led to an improvement in the user experience of the prototype. We noted an improvement in students’ rating of their user experience from the first cycle (3.8/5) to the final cycle (4.3/5). In addition, a Single Ease Question was administered to students following each task in the final cycle of testing. The results suggest that the Pathway prototype was considered relatively easy to use with an average score of 6.6 across all tasks. The thematic analysis of participants’ feedback produced eight themes under which participants’ feedback was summarised. These themes include (1) Pathway’s description, (2) anonymity and privacy, (3) WHO-5 well-being survey, (4) interface design, (5) user preferences, (6) recommendations page, (7) service details, and (8) students’ choice of services. Our findings indicate that students had clarity on their privacy needs and clear expectations about privacy policies. For instance, they wanted the privacy policy for Pathway to be presented in layman’s language. Students expressed an openness to completing the wellbeing survey, and they appreciated being directed to support services, even when they had a high wellbeing score. Participants liked the design of the prototype. They commented on the layout, language and colours. Participants felt that the questions in the preferences section were useful for those seeking support, and they made suggestions for the addition of preferences, for instance, location, and individual/group therapy. Further, participants noted that Pathway was distinct from other tools in the level of detail it provided about support services. They believed that this information would support students’ decision-making on services. Additionally, the inclusion of verified information and the citation of sources supported credibility and trust. Students believed that the Pathway app was more trustworthy than a web search. Finally, in comparing Pathway to other ways of searching for support, students noted that it was quicker, more personal and more trustworthy.

### 4.2 Relation to existing literature

Students had differing views on not needing to sign up to access Pathway, however, all students indicated that they appreciated this feature. This is unsurprising because previous research found that one of the reasons people stop using mobile apps is because of privacy issues, for instance, the need to create an account or an app asking for too much information during the login process [50].

Students were quite interested in completing the WHO-5 wellbeing survey. Some noted that even though it was optional, they wanted to complete the survey. Participants appreciated that the survey did not provide a diagnosis but instead helped them understand how they were feeling. This is similar to findings in a co-design study in which young adults designed an ideal online mental health resource to facilitate help-seeking [51]. Participants in the study believed it was important for a psychometric test to help “see where you’re at” instead of providing a diagnosis [51]. In addition, in this study, the survey results introduced a form of stepped care; participants mentioned that if they had a high well-being score, they would attend an informal service, and if they had a low well-being score, they would attend a formal service. Zivin et al. [52] suggests a form of stepped care in which students state their perceived level of need when accessing services. They noted that this could reduce the existing demand for mental health services, ensuring that those with greater need receive higher levels of support, e.g., through formal services [53]. The Pathway app presents an opportunity to provide stepped care by supporting students’ choices of support services [54] and reducing the burden on formal services [53].

Stakeholders had differing views on the question of preferred therapy approaches. However, they believed that promoting preferences regarding therapy would be empowering and help students understand what choices are appropriate. Previous research has identified improved mental health literacy as a facilitator of help-seeking in the student population [55]. This encompasses knowledge of available services, how to access support and information about the process of therapy [55]. Therefore, it is unsurprising that stakeholders recommend that students are taught about therapy and therapy approaches within the Pathway app. Consequently, it would be prudent to include an education feature within the Pathway app where students can learn about what to expect from therapy and the different types of therapy approaches, as this might encourage their use of services.

In this study, students indicated that they would prefer to use Pathway over their current methods of searching for mental health support, i.e., conducting a web search or asking friends. They believed that Pathway was quicker, more focused and trustworthy, and provided more privacy in finding appropriate support. They noted that searching for support using the internet would take time, and they might not get the support they need. This is consistent with other findings, such as the work of Pretorius et al. [51], which engaged young adults in finding mental health information for user personas using the internet. Participants who performed a Google search were overwhelmed by the amount of information returned, and they found it difficult to choose an appropriate option.

Previous research found that one of the barriers to online and offline help-seeking is uncertainty about confidentiality [55–57]. While exploring the website of a helpline, a participant in the usability study indicated that it was important for them to know how volunteers would keep their information private. This underscores the importance of including the privacy policies of support services within the Pathway app.

Prior work has reported on features that young adults believe are important for establishing credibility in an online resource [51, 58, 59]. Some of these features are testimonials from their peers [58], quotes from doctors and health professionals [58], endorsement from recognised institutions (e.g., a university, health institution or the Government) [51, 58], a link to local support services [51] and a reference to scientific data or authors [51]. In addition, they believed it was important to have information on how an online resource was developed, i.e., if it was backed by research and/or young adults were involved in the design [58, 59]. Finally, young adults believed that a visually appealing design and accessible layout were important indicators of credibility [51]. Participants in the usability study found the user interface and layout appealing and easy to use. The prototype included links to resources written by experts. At the beginning of the study, participants were informed that the prototype was based on the findings of a student survey. The sources of information were clearly stated and verification tags were used to signify information that was approved by mental health support services. Finally, the Pathway prototype included student reviews and links to local support services (including emergency services). Therefore, it is not surprising that students considered the Pathway app trustworthy.

In summary, participants in this study perceived the Pathway app as a useful and time-saving application that can help students find appropriate support. The app was described as simple and easy to learn. We believe that the content and usability requirements can serve as a guide for other researchers developing similar technologies [35]. Following the changes made in the study, the Pathway prototype now offers an improved design, making it better suited to support students in their search for mental health support services.

### 4.3 Contribution of pathway to the design of help-seeking technologies

As mentioned earlier, we know of three research-based help-seeking technologies that have documented end-user participation in the design process. In this section, we discuss what distinguishes Pathway from these technologies and how the input of stakeholders (including end-users) has guided the design of Pathway compared to these technologies.

Pathway distinguishes itself from previous tools by tailoring its content and design to the needs of the student population. For example, while ThoughtSpot [30] uses crowdsourcing to add services to the application, Pathway relies on support services to provide its information on the platform. This approach ensures credibility and trust, which is crucial because participants in a scoping review [40] identified the presence of trustworthy information in online interventions as important. Moreover, the Link prototype [16] and the website directory [25] were not designed for the student population who have distinct needs or circumstances, including the age of the cohort, moving away from home and engaging in unstructured education [60]. A technological tool is more likely to be successful (i.e., usable, accessible and enjoyable) if it considers the needs of its target population [24, 37].

The ThoughtSpot program allows users to find mental health resources through a map-based database [31]. Within ThoughtSpot, users can filter these services/resources based on languages, types of therapy, hours and availability, etc. Similarly, the web directory designed by Pretorius et al. [25] provides a variety of resources to users, allowing them to filter according to the category of a resource, for example, online chat, local services, personal stories, etc. The needs assessment that led to the design of Pathway helped us to understand that students had specific needs regarding mental health services that they wished to attend, including cost, mode of attendance (in-person or online), modality (e.g., phone calls, texts, chats, etc.) [35]. In addition, findings from our online survey indicate that students found the search for services to be overwhelming, even when they were browsing a support service website [61]. Therefore, we understood that listing resources without matching them to the users’ current level of need would not work for this population. Findings from the usability study indicate that this was the right approach. Participants noted that they were not overwhelmed, were satisfied with the questions asked and that those questions helped them consider what they would want in a service. It should be noted that these questions were selected based on the findings in the online survey [35]. In addition, unlike the ThoughtSpot program [31] and the web directory [25], Pathway includes a wellbeing survey within the app. The inclusion of the survey is based on a need identified within the survey, indicating that students felt that their difficulties were not “serious enough” for accessing mental health support [61]. This survey serves as an external validation of their needs. In addition, through the usability study, experts noted that the survey gives students language with which to express their difficulties. Further, students noted that the results of the survey supported them in their choice of services.

Participants in the ThoughtSpot usability study indicated that the program was not easy to figure out and that they had missed certain functionalities that were present within the app. Simplicity was one of the main priorities in the design of Pathway [35], and findings from the usability study indicate that participants found it easy to use and learn.

Following the usability study of the ThoughtSpot program, the authors noted that the needs identified during the co-design process did not reflect the needs of the population. One of the strengths of the Pathway app is that its design is based on students’ experiences seeking and accessing support as well as their experiences using online mental health resources [35, 61]. Although changes were made to the prototype during the usability tests, the needs that were identified were well reflected in the participants’ feedback.

Finally, the successful implementation of the Pathway app requires collaboration with mental health stakeholders. Through the usability study, we learned that stakeholders had concerns about student reviews and that these concerns might affect their collaboration with Pathway. This supports the importance of including stakeholders in the early design process of digital health technologies.

### 4.4 Strengths and limitations of this study

To account for the possibility of social desirability bias, the researcher (OO) told participants about the studies that led to the design of Pathway. The researcher emphasised the importance of their feedback in improving the prototype. They were encouraged to speak freely as the purpose of the study was to improve students’ access to mental health support services. Further, interview questions were open-ended to avoid leading participants to positive statements.

The students who participated in this study were from a single institution in Ireland, therefore, their feedback is not generalizable to the student population in Ireland and internationally. In addition, we have not targeted students with experience seeking support or who were currently searching for support. However, the inclusion of those with no experience seeking support helped us redesign the app from the perspective of potential first-time users of therapy. However, those with lived experience who are willing to discuss their experiences could further inform the research on the design of help-seeking technologies. Our findings should be interpreted in light of these limitations.

### 4.5 Future research

A majority of eHealth technologies are not adopted in practice, because stakeholders are often excluded from the design and development process of an intervention [41]. For instance, while students were happy with the reviews, other stakeholders expressed genuine concern. We need to establish a middle ground in which students’ need for credibility and connectedness is catered to, as well as stakeholders’ concern about reputational risk and the deterrent effect of negative reviews on help-seekers. Therefore, in our next study, we take a step towards implementation by conducting an in-depth evaluation of the Pathway app from the stakeholders’ perspective. We also explore challenges we might face when implementing the application in the student population. Further, as the Pathway prototype was in the early stage of development, we have not used standardised usability metrics such as the System Usability Scale. Instead of focusing on the cohesiveness of the system, we focused on the ease of completing tasks within the system. In a pilot study of the Pathway app, we will use more standard usability indicators, including task completion time, system usability scale and success rate. In addition, this pilot study will examine the effectiveness of Pathway in improving help-seeking intentions and behaviours among students. Finally, we will explore what type of survey feedback is more likely to encourage help-seeking in this population.

## 5 Conclusion

Searching for appropriate mental health support services can be overwhelming and time-consuming. The student population has a well-documented large treatment gap for mental health difficulties. Therefore, it is essential to support students in their search for support when they need it. Pathway provides an opportunity to connect students to the support they need in an easy and timely fashion.

## Data Availability

The original contributions presented in the study are included in the article/Supplementary Material, further inquiries can be directed to the corresponding author/s.
