# Supplementary material for: Usability testing of the Pathway app: engaging stakeholders to improve access to mental health support for students
Source: Front Digit Health. 2025 Nov 17;7:1633987. doi: 10.3389/fdgth.2025.1633987 (PMC12665652; doi:10.3389/fdgth.2025.1633987)
Supplement: Supplementary file 1 [file Datasheet1.pdf]

Usability Test Plan

Cycle 1 – Students

Starter task a: Navigate to the splash screen

Questions:

- The splash screen shows some information about what ‘Pathway’ does with your data - what do you understand by it? Is this something you would consider important to know? Why or why not?
- What would you want to see in the “Read Pathway’s privacy policies” link?

Starter task b: Navigate to the home screen.

- Based on the explanation of Pathway provided can you say “what this app is for and what you can do with it”?
- Is there some information you would want to be added or removed from here?
- Can you tell me what you think clicking on these 3 buttons will take you?

Task 1: Can you complete the mental health survey on Pathway, please select only “All of the time” as you go through the questions.

Scenario 1: Assuming that you have just taken the mental health survey on Pathway.

- How would you expect your results to be shown to you?
- How does it differ from the way Pathway presented it?

Scenario 2: You are a university student, and you want to get mental health support. Can you try to use Pathway to find a service? Can you tell me why you chose those options? In each of the questions?

- What do you think of the preferences you had to choose from?
- Would you want some preferences to be added or removed?
- Did you understand the options that were provided?

Scenario 3: ‘Pathway’ has presented a list of recommended services. What would you do next?

- How many services would you want to see after you have selected your preferences?
- In terms of a review, what would you want to know from students who have used the service?
- What do you think about the information provided on the services?
- Would you want less or more information?
- What kind of questions would you want to be answered in the FAQs?
- What criteria would guide your choice in selecting a service?

Scenario 4: Assuming that you have chosen to try out the service MyMind. What would you do next?

- Would you want Pathway to do more to help you access a service?
- Is there more information you would want about the service at this point?

Scenario 5: Supposing that you have attended the first counselling or therapy session and you feel you want to try a new service.

- How would you expect ‘Pathway’ to help?
- Would you want to enter your preferences a second time?
- Would you have expected ‘Pathway’ to save the earlier recommended services?

General Questions:

- How would you search for mental health support if you needed it?
- Can you compare this process to finding service through Pathway?
- Do you think that ‘Pathway’ would be useful to students looking for mental health support?
- Can you think of any features that would make students more likely to use ‘Pathway’?
- Can you rate your experience of using Pathway on a five-point scale, where 1 is very dissatisfied, and 5 is very satisfied?
- Can you tell me why you gave it a __ rating?

- Is there anything else you would like to say? e.g. about your experience with Pathway or about your experience seeking mental health support?

Cycle 1 – Experts (psychologists with experience in the design of mental health technology)

- Can you give your opinion on “Pathway” and its feasibility for a student seeking mental health support?
- Can you think of any features that would make students more likely to use ‘Pathway’?
- What do you think of the language used in Pathway especially when we give feedback to students on their mental health survey?
- Is there anything else you would like to say? About Pathway or about your experience with help-seeking among young people?

Cycle 2 – Stakeholders

What do you think of the Pathway application?

Cycle 3 – Students

Starter task a: Navigate to the splash screen

Starter task b: Navigate to the home screen.

- Based on the explanation of Pathway provided can you tell me what you think this app is for and what you can do with it?

Task 1: Can you complete the mental health survey using the Pathway app? Please select only “All of the time” as you go through the questions.

- Direct the participant to Single Ease Question
- If the score is below 5, ask “And can you tell me why you have rated it a ..”?

Post-task question

- Can you think of a better way of presenting the results?
- How would you expect it to be presented to you?
- If you had a low well-being score (below 50), how would you expect the results to be presented to you?

Scenario: You are a university student, and you want to get mental health support.
Task 2: Can you use Pathway to find a suitable service?

- Direct the participant to Single Ease Question
- If the score is below 5, ask "And can you tell me why you have rated it a .."?

Post-task question

- Ask a follow-up question from their think-aloud – if they don't say anything, ask what they were thinking as they were completing the task

Scenario: 'Pathway' has presented a list of recommended services.
Task 3: Can you use Pathway to find information to support your decision-making process for the service MyMind/Samaritans?

- Direct the participant to Single Ease Question
- If the score is below 5, ask "And can you tell me why you have rated it a .."?

Post-task question

- Did you feel that you had enough information from Pathway to decide that this service is the right one for you?

Scenario: Now that you have chosen to try out MyMind/Samaritans

- What is your next step?

Scenario: Supposing that you have attended the first counselling or therapy session and you feel you want to try a different service.
Task 4: Can you use Pathway to find a new service?

- Direct the participant to Single Ease Question
- If the score is below 5, ask "And can you tell me why you have rated it a .."?

General Questions:

- In the absence of Pathway, how would you search for mental health support if you needed it?
- Can you compare this process to finding service through Pathway?
- Can you rate your experience of using Pathway on a five-point scale, where 1 is very dissatisfied, and 5 is very satisfied?
- Can you tell me why you gave it a __ rating?

- Is there anything else you would like to say? e.g. About your experience with Pathway or about your experience seeking mental health support?
